# Role of SAMHD1 nuclear localization in restriction of HIV-1 and SIVmac

**DOI:** 10.1186/1742-4690-9-49

**Published:** 2012-06-12

**Authors:** Alberto Brandariz-Nuñez, Jose Carlos Valle-Casuso, Tommy E White, Nadine Laguette, Monsef Benkirane, Jurgen Brojatsch, Felipe Diaz-Griffero

**Affiliations:** 1 Department of Microbiology and Immunology, Albert Einstein College of Medicine Bronx, Bronx, NY, 10461, USA; 2 Laboratoire de Virologie Moléculaire, Institut de Génétique Humaine, CNRS-UPR 1142, 141 rue de la Cardonille, 34296, Montpellier, cedex 5, France

**Keywords:** SAMHD, Restriction, Nuclear localization, Vpx, HIV-1, SIV, Degradation

## Abstract

**Background:**

SAMHD1 is a nuclear protein that blocks lentiviral infection before reverse transcription in macrophages and dendritic cells. The viral accessory protein Vpx overcomes the SAMHD1-mediated lentiviral block by inducing its proteasomal degradation.

**Results:**

Here, we identified the nuclear localization signal (NLS) of SAMHD1, and studied its contribution to restriction of HIV-1 and SIVmac. By studying the cellular distribution of different SAMHD1 variants, we mapped the nuclear localization of SAMHD1 to residues ^11^KRPR^14^. Mutagenesis of these residues changed the cellular distribution of SAMHD1 from the nucleus to the cytoplasm. SAMHD1 mutants that lost nuclear localization restricted HIV-1 and SIV as potently as the wild type protein. Interestingly, SAMHD1 mutants that localized to the cytoplasm were not degraded by nuclear Vpx alleles. Therefore, nuclear Vpx alleles require nuclear localization of SAMHD1 in order to induce its degradation. In agreement, SIVmac viruses encoding Vpx did not overcome the restriction imposed by the cytoplasmic variants of SAMHD1.

**Conclusions:**

We mapped the NLS of SAMHD1 to residues ^11^KRPR^14^ and studied the contribution of SAMHD1 nuclear localization to restriction of HIV-1 and SIV. These experiments demonstrate that cytoplasmic variants of SAMHD1 potently block lentiviral infection and are resistant to Vpx-mediated degradation. The nuclear Vpx alleles studied here are only capable of degrading a nuclearly localized SAMHD1 suggesting that Vpx-mediated degradation of SAMHD1 is initiated in the nucleus.

## Background

Infection of primary macrophages and dendritic cells by Simian Immunodeficiency virus (SIV) requires the accessory protein Vpx, which is encoded in the SIV genome [1]. SIV particles without Vpx (SIVΔVpx) are unable to infect primary macrophages. Vpx is essential for the ability of SIV to infect primary macrophages *in vitro* and for viral dissemination and pathogenesis *in vivo*[2-5]. Vpx is incorporated into viral particles suggesting that it might be acting immediately after viral fusion with target cells [6-9]. Viral reverse transcription is prevented in primary macrophages when cells are infected with Vpx-deficient SIV or HIV-2 [10-14]. These experiments suggest that when Vpx is incorporated into viral particles, the virus overcomes a pre-reverse transcription block in macrophages and dendritic cells. Remarkably, Vpx also increases the ability of HIV-1 to infect macrophages and dendritic cells when Vpx is incorporated into HIV-1 particles or supplied in *trans*[15,16]. This suggests that the block imposed by macrophages to SIVΔVpx is similar to the one imposed by macrophages to HIV-1.

Recent work identified SAMHD1 as the protein that blocks infection of SIVΔVpx and HIV-1 before reverse transcription in macrophages and dendritic cells [17,18]. Mutations in SAMHD1 cause Aicardi-Goutières syndrome, which is a genetic disease that causes encephalopathy closely resembling sequelae from congenital infection [19]. SAMHD1 contains a sterile alpha motif (SAM) and a histidine-aspartic (HD) domain. SAM domains are protein interaction modules that mediate interaction with other SAM domains or non-SAM domain-containing proteins [19]. Additionally, SAM domains in other proteins bind a specific sequence of DNA acting as transcription activators or repressors [20]. The HD domain is found in a super family of enzymes with a predicted phosphohydrolase activity [21]. In agreement, recent work has demonstrated that SAMHD1 is a dGTP-regulated deoxynucleotide triphosphohydrolase that might be involved in decreasing the overall cellular levels of triphosphodeoxynucleotides [22-24].

SAMHD1 localizes to the nucleus in human fibroblasts as demonstrated by subcellular localization studies either using antibodies against the endogenous protein or the study of a GFP-SAMHD1 fusion construct [19]. Mechanistic studies have suggested that Vpx induces the proteasomal degradation of SAMHD1[17,18]. In addition, localization studies of the Vpx protein during SIV or HIV-1 infection correlated the nuclear localization of Vpx with its ability to promote productive infection in primary macrophages [5,15,25,26], suggesting that Vpx might overcome the viral-block imposed by SAMHD1 in the nucleus. Therefore, we decided to study the role of SAMHD1 nuclear localization in its ability to block HIV-1 and SIVmac infection. For this purpose, we first identified the NLS of SAMHD1 and analyzed the ability of SAMHD1 NLS variants to block infection. In addition, we tested the ability of Vpx to induce the degradation of SAMHD1 NLS variants.

## Results

### SAMHD1 is a nuclear protein

Endogenous SAMHD1 localizes to the nuclear compartment of human MRC-5 fibroblasts [19]. Here, we tested the localization of endogenous SAMHD1 in monocyte-derived macrophages (MDM), PMA-differentiated THP-1 cells, and HeLa cells. In addition, we studied the localization of epitope tagged exogenous SAMHD1 in HeLa cells. As previously reported, in all cell types studied here, endogenously and exogenously expressed SAMHD1 localized to the nuclear compartment Additional file ( 1A-C). Interestingly, SAMHD1 did not co-localized with fibrilarin suggesting that SAMHD1 is in the nucleoplasm, but not in the nucleolus Additional file ( 1D). Overall these results indicate that SAMHD1 localizes to the nucleus, and that the cytoplasm contains a very small fraction of the total amount of SAMHD1 in the cell. To understand the contribution of SAMHD1 nuclear localization to restriction of HIV-1 and SIVmac, we first investigated the region of SAMHD1 that contributes to its nuclear import.

**Table 1:** Immunostaining data

**SAMHD1**	**Experiment 1**			**Experiment 2**			**Experiment 3**			**Figure**
	**Exclusively nuclear**	**Exclusively cytoplasm**	**Throughout cell**	**Exclusively nuclear**	**Exclusively cytoplasm**	**Throughout cell**	**Exclusively nuclear**	**Exclusively cytoplasm**	**Throughout cell**	
GFP-SAMHD1	184	0	16	190	0	10	188	0	12	1B
GFP-SAMHD1 (1–328)	176	0	24	180	0	20	177	0	23	1B
GFP-SAMHD1(329-626	0	40	160	0	46	154	0	38	162	1B
GFP-SAMHD1(1–150)	156	0	44	160	0	40	164	0	36	1B
GFP-SAMHD1(151–328)	0	0	200	0	0	200	0	0	200	1B
GFP-SAMHD1 K11A	0	188	12	0	190	10	0	192	8	2B
GFP-SAMHD1 R12A	0	190	10	0	194	6	0	188	12	2B
GFP-SAMHD1 R14A	0	192	8	0	196	4	0	186	14	2B
SAMHD1-FLAG	196	0	4	194	0	6	196	0	4	2D
SAMHD1-FLAG K11A	0	194	6	0	192	8	196	0	4	2D
SAMHD1-FLAG R12A	0	196	4	0	194	6	0	192	4	2D
SAMHD1-FLAG R14A	0	192	8	0	190	10	0	188	12	2D
GFP-muNS	0	200	0	0	200	0	0	200	0	3A
KRPR-GFP-muNS	150	0	50	146	0	54	152	0	48	3A
GFP	0	0	200	0	0	200	0	0	200	3B
KRPR-GFP	140	0	60	146	0	54	142	0	58	3B
SAMHD1-FLAG (U937)	194	0	6	190	0	10	196	0	4	4C
SAMHD1-FLAG K11A (U937)	194	0	6	190	0	10	196	0	4	4C
SAMHD1-FLAG R12A (U937)	0	192	8	0	196	4	0	188	14	4C
SAMHD1-FLAG R14A (u937)	0	190	10	0	184	16	0	186	14	4C
SAMHD1 (MDM)	196	0	4	192	0	8	198	0	2	AF1A
SAMHD1 (THP1 + PMA)	198	0	2	196	0	4	200	0	0	AF1A
SAMHD1 (Endogenous in Hela)	194	0	6	200	0	0	196	0	4	AF1A
SAMHD1 (Tramsient expression)	198	0	2	200	0	0	194	0	6	AF1B
SAMHD1 (Stable expression	196	0	4	196	0	4	200	0	0	AF1B
SAMHD1-FLAG (15–626)	0	194	6	0	196	4	0	190	10	AF2A

Three independent experiments were performed. Cells were scored visually for nuclear and cytoplasmic distribution. In every experiment two hundred cells were counted. The analysis of the distribution was performed in human HeLa cells or otherwise indicated. MDM = Monocyte derived macrophages. PMA = phorbol-12-myristate-13-acetate.

### The nuclear localization signal (NLS) of SAMHD1 is contained in the first 150 amino acids

**Figure 1:**
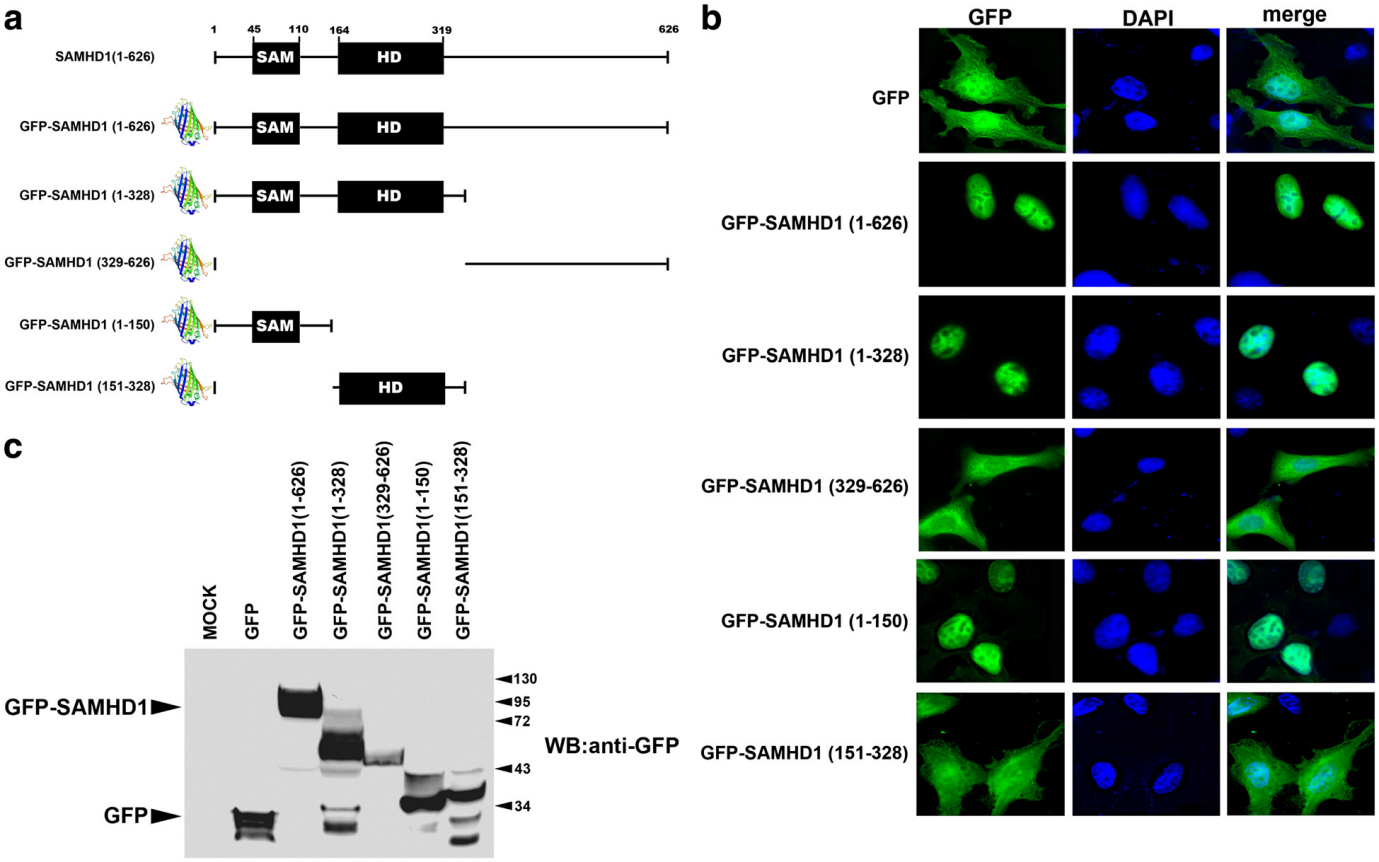
**The nuclear localization signal of SAMHD1 is contained in the first 150 amino acids.** (**A**) Schematic representation of the different SAMHD1 GFP-fusion constructs. Full-length SAMHD1 is schematically represented, and the numbers of the amino acids residues at the boundaries of the protein domains are shown. The N terminus of the protein is fused to GFP, which is shown as its own structure. (**B**) HeLa cells expressing the indicated GFP-fusion constructs (green) were imaged by fluorescence microscopy. The cellular nuclei were stained by using DAPI (blue). Image quantification for three independent experiments is shown in Table 1. (**C**) Expression of SAMHD1 GFP-fusion constructs was analyzed by Western blotting using antibodies against GFP. The different constructs are indicated and the molecular weight markers are shown. Similar results were obtained in three independent experiments and a representative experiment is shown.

Protein transport from the cytosol to the nucleus generally requires the existence of a NLS [27]. To test for the presence of an NLS in SAMHD1, we fused the green fluorescent protein (GFP) to different regions of the SAMHD1 protein (Figure 1A) and tested the different constructs for nuclear localization and expression in HeLa cells (Figure 1B-C).

Our mapping studies revealed that SAMHD1 contains a putative NLS in the first 150 amino acids (Figure 1). The GFP fusion with the full-length SAMHD1 [GFP-SAMHD1(1-626)] localized to the nucleus similar to the endogenously expressed protein (Figure 1B). In contrast to the fusion GFP-SAMHD1(329-626) that contains the C-terminal half of the protein, GFP-SAMHD1(1-328) exclusively localized to the nucleus (Figure 1B), suggesting that the putative NLS is located in the first 328 amino acids. To further narrow the location of a potential NLS, we tested GFP-SAMHD1(1-150) and GFP-SAMHD1(151-328). Contrary to GFP-SAMHD1(151-328), the GFP fusion construct that contains the first 150 amino acids of SAMHD1 exclusively localized to the nucleus [GFP-SAMHD1(1-150)]. These results suggested that the sequence required for nuclear localization of SAMHD1 is comprised within the first 150 amino acids, which is in agreement with the nuclear localization of a naturally occurring truncation of SAMHD1 that only contains the first 149 amino acids [19].

### Site-directed mutagenesis of the putative SAMHD1 NLS

**Figure 2:**
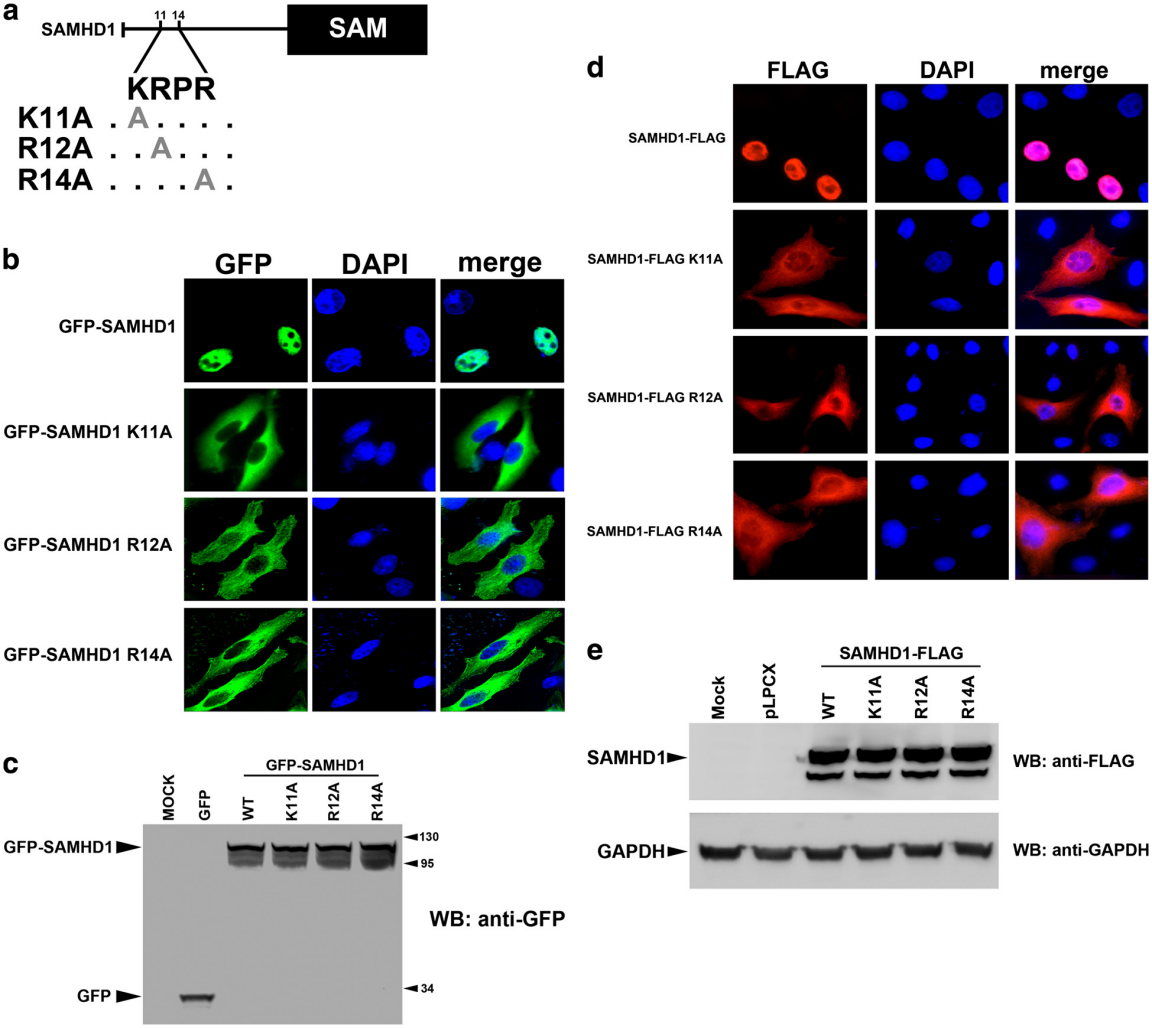
**Identification of**^**11**^**KRPR**^**14**^**as the functional SAMHD1 nuclear localization signal.** (**A**) Diagram showing the amino acid substitutions in the putative nuclear localization signal of SAMHD1. (**B**) HeLa cells expressing the indicated GFP-SAMHD1 variant were imaged by fluorescence microscopy(green). The cellular nuclei were stained by using DAPI (blue). Image quantification for three independent experiments is shown in Table 1. (**C**) The expression of GFP-SAMHD1 variants in HeLa cells was measured by Western blotting using antibodies against GFP. (**D**) HeLa cells expressing the indicated SAMHD1-FLAG variants were fixed and immunostained using antibodies against FLAG (red). Similarly, cellular nuclei were stained by using DAPI(blue). Image quantification for three independent experiments is shown in Table 1. (**E**) Expression of the indicated SAMHD1-FLAG variants in HeLa cells was analyzed by Western blotting using antibodies against FLAG. As a loading control, cell lysates were Western blotted using antibodies against GAPDH. Similar results were obtained in three independent experiments and a representative experiment is shown.

Analysis of the first 150 amino acids of SAMHD1 revealed that the peptide ^11^KRPR^14^ starting at position 11 is a potential NLS (Figure 2A) [28]. To test this hypothesis, we mutagenized the potential NLS in the GFP-SAMHD1(1-626) construct and tested these mutants for subcellular localization and expression (Figure 2B-C). All tested GFP-SAMHD1(1-626) mutants of the ^11^KRPR^14^ peptide localized to the cytoplasmic compartment (Figure 2B). Thus, the ^11^KRPR^14^ sequence may act as nuclear localization signal of SAMHD1. We ruled out the potential influence of the GFP in the localization of SAMHD1 by performing similar changes in a FLAG-tagged SAMHD1 construct. We tested the subcellular localization and expression of the FLAG-tagged SAMHD1 variants (Figure 2D-E). Similarly, we found that changes in the NLS localized SAMHD1 to the cytoplasm. To confirm these results, we also tested the localization of a deletion construct of SAMHD1 that does not contain the first 14 amino acids. In agreement with our findings that the initial 14 amino acids of SAMHD1 contain the NLS, the construct SAMHD1(15-626)-FLAG localized exclusively to the cytoplasm Additional file ( 2). Next, we tested whether this NLS is sufficient to target unrelated proteins to the nucleus.

### The nuclear localization signal of SAMHD1 is transferable

**Figure 3:**
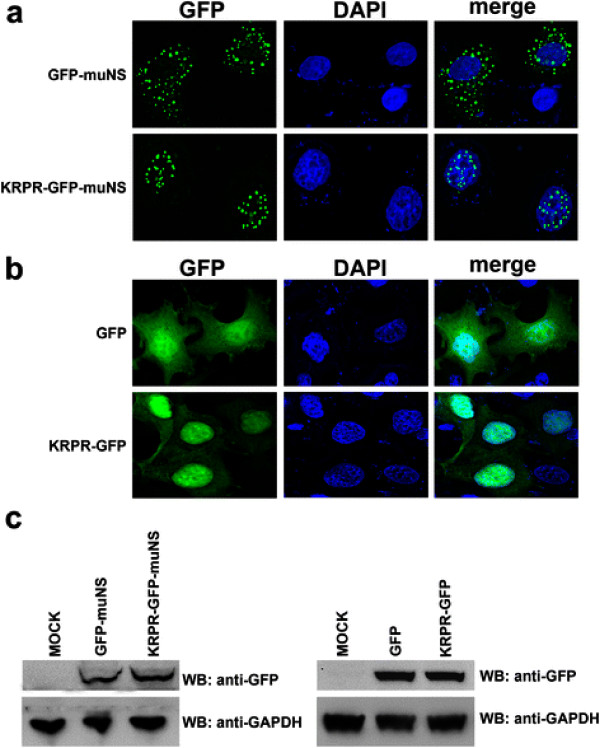
**The nuclear localization signal of SAMHD1 is transferable.** (**A**) HeLa cells expressing a fusion between the NLS of SAMHD1 and the cytoplasmic GFP-muNS protein (KRPR-GFP-muNS) were imaged by fluorescence microscopy (green). As a control, the cytoplasmic distribution of the GFP-muNS protein is shown. Cellular nuclei were stained by using DAPI (blue). Image quantification for three independent experiments is shown in Table 1. (**B**) Similarly, HeLa cells expressing a fusion between the NLS of SAMHD1 and GFP (KRPR-GFP) were imaged by fluorescence microscopy(green). Cellular nuclei were stained by using DAPI (blue). Image quantification for three independent experiments is shown in Table 1. (**C**) The expression of the indicated fusion proteins was analyzed by Western blotting using antibodies against GFP. As loading controls, cell lysates were Western blotted using antibodies against GAPDH. Similar results were obtained in three independent experiments and a representative experiment is shown.

To test whether the NLS of SAMHD1 is transferable, we incorporated the NLS of SAMHD1, ^11^KRPR^14^, to the N terminus of a GFP protein fused to the viral protein muNS (GFP-muNS) from avian reovirus that localizes to the cytoplasm (Figure 3A) [29,30]. Remarkably, the construct expressing KRPR-GFP-muNS was exclusively localized to the nuclear compartment (Figure 3A, C). This suggests that the SAMHD1 NLS is sufficient to change the localization of cytosolic GFP-muNS. Similar results were obtained by fusing the NLS of SAMHD1 to a wild type GFP protein (Figure 3B, C). Overall these results demonstrated that the NLS of SAMHD1, ^11^KRPR^14^, is sufficient to allow the import of an unrelated protein to the nucleus. Thus, the ^11^KRPR^14^ sequence is the nuclear localization signal of SAMHD1.

### Contribution of SAMHD1 nuclear localization to restriction of HIV-1

**Figure 4:**
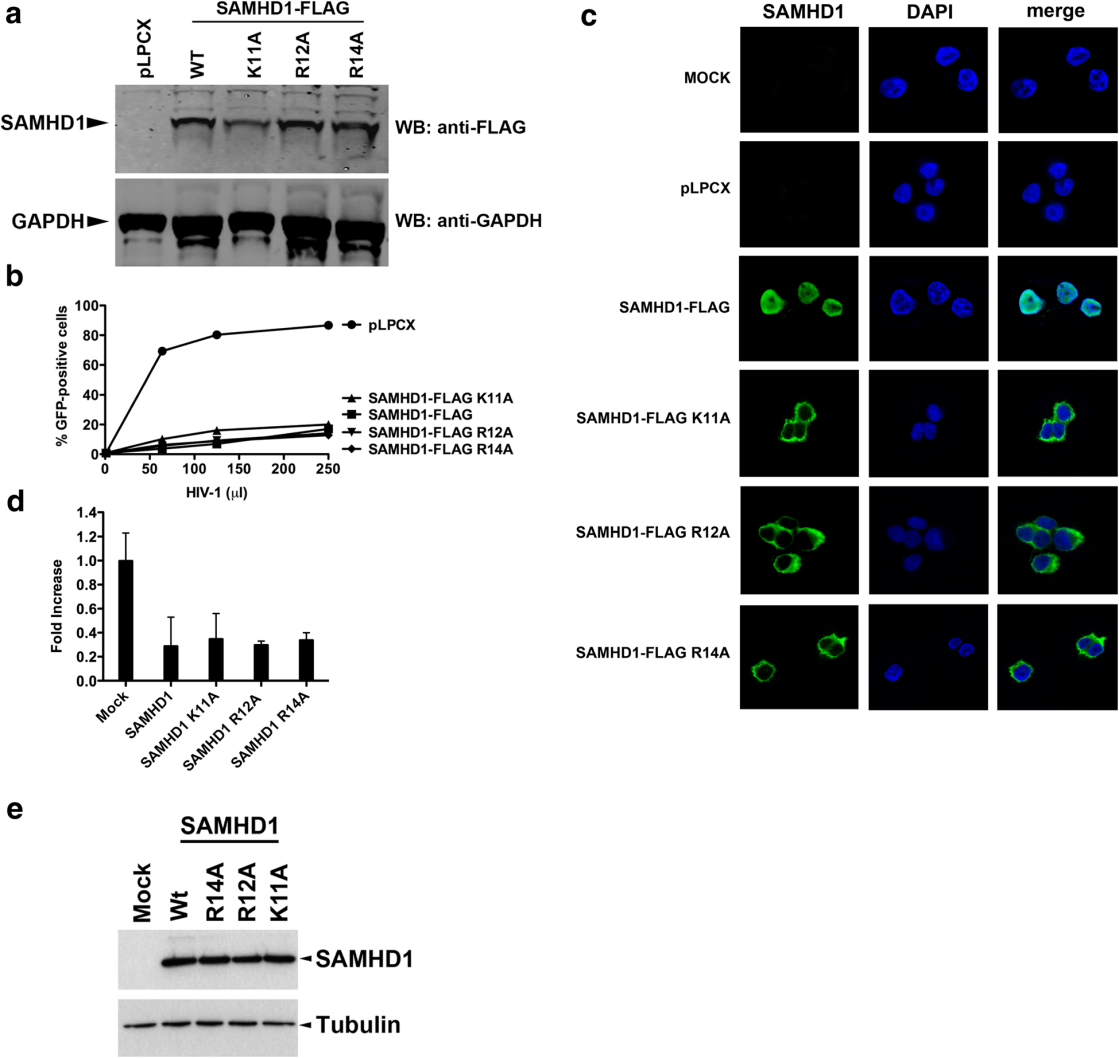
**Contribution of SAMHD1 nuclear localization to restriction of HIV-1.** Human monocytic U937 cells stably expressing the indicated mutant and wild type SAMHD1 proteins (**A**) were challenged with increasing amounts of HIV-1-GFP (**B**). As a control, U937 cells stably transduced with the empty vector LPCX were challenged with HIV-1-GFP. (**C**) U937 cells stably expressing the indicated SAMHD1-FLAG variants were fixed and immunostained using antibodies against SAMHD1 (green). The cellular nuclei were stained by using DAPI (blue). Mock represents wild type U937 cells. Image quantification for three independent experiments is shown in Table 1. (**D**) SAMHD1-silenced THP-1 cells were transiently transduced with the indicated wild type and mutant SAMHD1 proteins. Forty-eight hours post-transduction cells were differentiated and infected with HIV-1 LUC-G, which expresses luciferase as a reporter for infection. Luciferase activity was measured 24 hours post-infection. Results are expressed as fold increase luciferase activity in transduced over mock THP-1 cells. (**E**) The level of mutant and wild type SAMHD1 expression, in transduced SAMHD1-silenced THP-1 cells, was analyzed by Western blotting using anti-FLAG antibodies. As loading control, we analyzed cell extracts by Western blot using anti-tubulin antibodies. Similar results were obtained in three independent experiments and a representative experiment is shown.

The nuclear localized SAMHD1 blocks retroviral infection before reverse transcription [10-14]. Here, we wanted to test whether nuclear localization of SAMHD1 is required for restriction of HIV-1. To test for this requirement, we challenged human monocytic U937 cells stably transduced with wild type and mutant SAMHD1 proteins with increasing amounts of HIV-1 (Figure 4A, B). The SAMHD1 NLS mutants that localized to the cytoplasm restricted HIV-1 as efficiently as the wild type protein (Figure 4B, C). Similar HIV-1 restriction was observed when expressing the different SAMHD1 variants in THP-1 cells that were stably silenced for expression of endogenous SAMHD1 (Figure 4D, E). Altogether these results indicated that restriction of HIV-1 by SAMHD1 does not require the presence of SAMHD1 in the nucleus.

### Vpx-mediated degradation of SAMHD1 requires nuclear localization of SAMHD1

**Figure 5:**
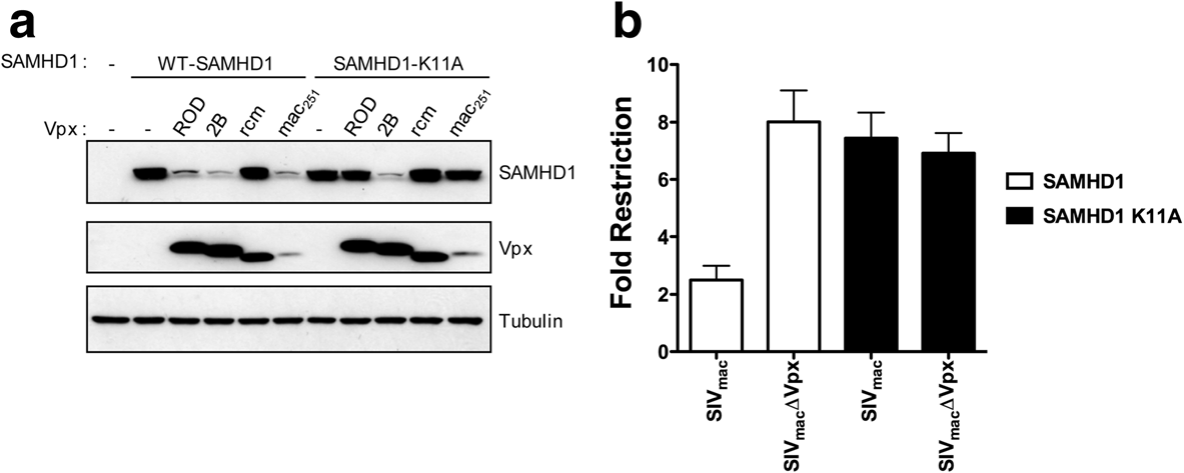
**Vpx**_**n**_**-induced degradation of SAMHD1 requires nuclear localization of SAMHD1.** (**A**) HeLa cells were cotransfected with plasmids allowing expression of SAMHD1-FLAG or SAMHD1-K11A-FLAG and HA-tagged Vpx from SIVmac 251(Vpx_mac251_), SIVrcm-ng (Vpx_rcm-ng_), HIV-2ROD (Vpx_ROD_) or HIV-2B (Vpx_2B_). Thirty-six hours post-transfection the cells were harvested, and the expression levels of SAMHD1 and Vpx were analyzed by Western blot using anti-FLAG and HA antibodies. As a loading control, cell extracts were Western blotted using antibodies against Tubulin. Similar results were obtained in three independent experiments and a representative experiment is shown. (**B**) Human monocytic U937 cells stably expressing the indicated mutant or wild type SAMHD1 proteins were challenged with SIVmac or SIVmacΔVpx -GFP reporter viruses. Infection was determined by measuring the percentage of GFP-positive cells. Fold of restriction was calculated by dividing the % of GFP-positive cells resulting from infecting U937 cells containing the empty vector pLPCX by the % GFP-positive cells resulting from infecting U937 stably expressing the indicated SAMHD1 variant. Similar results were obtained in three independent experiments and standard deviation is shown.

The SIV accessory protein Vpx overcomes SAMHD1 restriction by triggering its degradation [17,18]. As shown, SAMHD1 is localized to the nuclear compartment when endogenously or exogenously expressed [19]. In agreement, localization studies of the Vpx protein during lentiviral infection correlated the nuclear localization of Vpx with its ability to promote productive infection in primary macrophages [5,15,25,26], which suggests that Vpx might overcome the viral-block imposed by SAMHD1 in the nucleus. We hypothesized that nuclear localization of SAMHD1 is required for the ability of Vpx to induce its degradation and overcome restriction. For this purpose, we measured the ability of nuclear and cytoplasmic Vpx proteins to induce degradation of SAMHD1 variants that localized to the cytoplasm (Figure 5A). In agreement with our hypothesis, the nuclear alleles of Vpx: Vpx from the ROD strain of HIV-2 (Vpx_ROD_) and Vpx from SIVmac_251_ (Vpx-mac_251_) Additional file ( 3) [31], did not trigger the degradation of the cytoplasmic variant SAMHD1-K11A (Figure 5A). As expected, Vpx_ROD_ and Vpx-mac_251_ potently degraded the nuclearly localized wild type SAMHD1 [31]. Remarkably, a cytoplasmic allele of Vpx, Vpx from HIV-2B (Vpx_2B_) Additional file ( 3) [31], induced degradation of the cytoplasmic SAMHD1-K11A (Figure 5A). As controls, we used the Vpx protein from SIV_Rcm-ng_ (Vpx_Rcm-ng_) that is unable to induce degradation of mutant and wild type SAMHD1 [31]. Next, we tested the ability of SIVmac to overcome the restriction imposed by SAMHD1 (Figure 5B). In agreement with our results that Vpx from SIVmac is unable to trigger the degradation of SAMHD1-K11A, we found that SIVmac is not capable of overcoming the restriction imposed by SAMHD1-K11A (Figure 5B). As expected, SIVmacΔVpx did not overcome the restriction imposed by any of the tested SAMHD1 variants (Figure 5B). These experiments suggested that Vpx nuclear alleles require nuclearly localized SAMHD1 in order to induce its degradation and to overcome restriction.

The observation that the different cytoplasmic variants of SAMHD1 described in this work blocked HIV-1 infection suggested that restriction does not require nuclear localization of SAMHD1. Therefore, we tested whether SAMHD1 changes localization upon HIV-1-GFP infection. For this purpose we infected PMA-treated U937 cells stably expressing SAMHD1 or SAMHD1-K11A with HIV-1. At different hours post-infection, samples were fixed, and the localization of SAMHD1 was determined Additional file ( 4). Even though PMA-treated U937 cells stably expressing SAMHD1 or SAMHD1-K11A potently restricted HIV-1 infection Additional file ( 4), we did not observe changes in the subcellular localization of SAMHD1.

## Discussion

Here, we mapped the nuclear localization signal of the restriction factor SAMHD1 and evaluated the impact of SAMHD1 cellular distribution on restriction of HIV-1 and SIVmac. Remarkably, mutations in the NLS of SAMHD1 that caused localization of the protein to the cytosol did not affect the ability of SAMHD1 to block HIV-1 or SIV infection. These results imply that SAMHD1 localization to the cytoplasm is sufficient to restrict HIV-1 infection. Because wild type SAMHD1 is mainly localized to the nucleus [19], taken together these observations suggested that SAMHD1 can deplete the overall cellular pool of deoxynucleoside triphosphates when in nucleus or in the cytosol [22-24]. An alternative possibility is that SAMHD1 is recruited to the cytoplasm in order to deplete the cellular pool of deoxynucleoside triphosphates; however, we did not observe changes on the cellular localization of SAMHD1 when cells were challenged by HIV-1 Additional file ( 4).

While revising this manuscript, mutations on SAMHD1 (I201N and M254V) that partially localized to the cytosol were described, suggesting that these residues might be contributing to the nuclear localization of SAMHD1[32]. However, due to the limited available data on mutations I201N and M254V, it is difficult to conclude whether these residues have a contribution to the nuclear import of SAMHD1. Current issues include: 1) the absence of functional data for mutants I201N and M254V other than subcellular localization [32], 2) residues I201 and M254 are not surface-exposed in the recently described structure of the HD domain[24], and 3) absence of evidence to demonstrate that these residues are part of a transferable NLS [32]. Future experiments will attempt to test whether I201 and M254 residues contribute to the nuclear localization of SAMHD1.

Wild type SAMHD1 localizes to the nucleus. In agreement, localization studies of the Vpx protein during SIV infection correlated the nuclear localization of Vpx with its ability to promote productive infection in primary macrophages [5,15,25,26]. Overall, this suggests that Vpx encounters SAMHD1 in the nucleus; therefore, Vpx modulation of SAMHD1 levels might start in the nuclear compartment. To test this hypothesis, we assayed the ability of nuclear localized Vpx proteins (Vpx_n_) to degrade SAMHD1 when in the nucleus or cytosol. Remarkably, Vpx_n_ only degraded nuclear SAMHD1. By contrast, SAMHD1 variants that localized to the cytoplasm were not affected by Vpx_n._ These results suggested that Vpx_n_-induced degradation of SAMHD1 begins when both proteins are in the nucleus. In agreement, SIV encoding Vpx_n_ was unable to overcome the block imposed by cytoplasmic variants of SAMHD1. These results contribute to our understanding of the mechanism used by Vpx_n_ to overcome the lentiviral block imposed by SAMHD1.

The fact that Vpx_n_ degrades only nuclear localized SAMHD1 suggests that this encounter is the first step of the degradation process of SAMHD1. The second step might be the export of SAMHD1 to the cytoplasm, as suggested by experiments in which leptomycin B blocked the Vpx_n_-induced degradation of SAMHD1 [33]. The final step is likely to be the cytoplasmic proteasomal degradation of SAMHD1 [10,11,15,34].

SAMHD1 restriction of lentiviruses might occur by a direct or an indirect mechanism. A direct mechanism would imply that SAMHD1 recognizes a specific element in the restricted virus; this further suggests the existence of a viral determinant for restriction. Elegant cross-packaging experiments using HIV and SIV demonstrated that dendritic cells and macrophages are more susceptible to an HIV-1 reporter RNA packaged by an HIV-1 Gag-Pol when compared to an SIV reporter RNA packaged with HIV-1[35]. These experiments suggest that the viral determinant for the block observed in dendritic cells and macrophages is the viral RNA. This is in agreement with the idea that SAMHD1 blocks retroviral infection before reverse transcription. Future experiments will attempt to identify whether the block observed by cross-packaging experiments in dendritic cells and macrophages is related to SAMHD1.

An indirect mechanism would imply that the enzymatic activity of SAMHD1 is sufficient to restrict HIV-1 replication. Recent work has demonstrated that the triphosphohydrolase activity of the HD domain of SAMHD1 is involved in decreasing the overall cellular levels of deoxynucleoside triphosphates and causing the restriction [22-24]. Depletion of deoxynucleoside triphosphates might occur either in the vicinity or far from where the virus is undergoing reverse transcription.

## Conclusions

We found that the nuclear localization signal of SAMHD1 mapped to residues ^11^KRPR^14^. The nuclear localization of SAMHD1 is important for the ability of Vpx to trigger degradation of SAMHD1. This work showed that cytoplasmic variants of SAMHD1 potently block HIV-1 and SIV infection and are resistant to Vpx_n_. The Vpx_n_ alleles studied here are only capable of degrading a nuclearly localized SAMHD1 suggesting that Vpx-mediated degradation of SAMHD1 is initiated in the nucleus.

## Methods

### Plasmids

The pEGFP-C1-M3 vector, which expresses full-length avian reovirus muNS non-structural protein, was a generous gift from Dr. Jose Martínez-Costas (University of Santiago de Compostela), and has been described previously [30]. The plasmids pLPCX-SAMHD1 and pLPCX-SAMHD1-FLAG expressing SAMHD1 and SAMHD1 fused to a FLAG epitope, respectively, were codon optimized by GENEWIZ. For the expression of fusions with green fluorescent protein (GFP), SAMHD1 sequences were amplified by PCR, digested with appropriate restriction enzymes, and inserted into the pEGFP-C1 vector (Clontech). To generate inserts for the production of pEGFP-C1 recombinant plasmids, we used the following primers: for the production of GFP-SAMHD1 (1-626), the sense primer was 5′-GCGGAATTCTATGCAGAGAGCTGATAGCG-3′ and the reverse primer was 5′-GCGGGATCCTCACATAGGGTCGTCC-3′; for the production of GFP-SAMHD1(1-328), the sense primer was 5′-GCGGAATTCTATGCAGAGAGCTGATAGCG -3′ and the reverse primer was 5′-GCGGGATCCTCAGTTATTCTGAATGCCCAGG -3′; for the production of GFP-SAMHD1 (329-626), the sense primer was 5′-GCGGAATTCTTTCGATTACAAAAGGTTTATC-3′ and the reverse primer was 5′-GCGGGATCCTCACATAGGGTCGTCC-3′; for the production of GFP-SAMHD1 (1-150), the sense primer was 5′- GCGGAATTCTATGCAGAGAGCTGATAGCG -3′ and the antisense primer was 5′-GCGGGATCCTCACAGCTGCTTGATATATCTG-3′, and for the production of GFP-SAMHD1 (151-328), the sense primer was 5′-GCG GAA TTC T GGC GGA GGC TAC TAT GTC -3′ and the reverse primer was 5′- GCGGGATCCTCAGTTATTCTGAATGCCCAGG-3′. The correct orientation of the inserts was confirmed by sequencing and restriction analysis.

To express the SAMHD1 NLS fused to the N terminus of GFP-muNS the recombinant plasmids pEGFP-C1-M3 [30] was subjected to PCR amplification with the following primers: the forward primer was 5′-GCGGGATCCACCATGGGAAAGAGACCCAGGACCATGGTGAGCAAGGGCGAG-3, and the reverse primer was 5′-GCGTCTAGATTACAGATCATCCACCAATTCTTC-3′. PCR products were digested and cloned into the BamHI and XbaI sites of pCDNA3.1/Zeo.

To express the SAMHD1 NLS fused to the N terminus of GFP the plasmid pEGFP-C1 (Clontech) was subjected to PCR amplification with the following primers: the forward primer was 5′-GCGGGATCCACCATGGGAAAGAGACCCAGGACCATGGTGAGCAAGGGCGAG-3, and the reverse primer was 5′-GCGTCTAGATTACTTGTACAGCTCGTCCATGCC-3′. PCR products were digested and cloned into the BamHI and XbaI sites of pCDNA3.1/Zeo.

The pLPCX-SAMHD1-FLAG plasmid, the pEGFP-SAMHD1 plasmid and a QuikChange site-directed mutagenesis kit (Stratagene) were used according to the manufacturer’s specifications to generate recombinant plasmids that expressed SAMHD1 variants K11A, R12A and R14A. The following mutagenic oligonucleotide primers were used: for the production of mutation K11A, the sense primer was 5′-GATAGCGAGCAACCCAGCGCGAGACCCAGGTGCGACG-3′ and the antisense primer was 5′-GTCGCACCTGGGTCTCGCGCTGGGTTGCTCGCTATC-3′; for the production of mutation R12A, the sense primer was 5′-GATAGCGAGCAACCCAGCAAGGCACCCAGGTGCGACG-3′ and the antisense primer was 5′-CGTCGCACCTGGGTGCCTTGCTGGGTTGCTCGCTATC-3′; and for the production of the mutation R14A, the sense primer was 5′-CCCAGCAAGAGACCCGCGTGCGACGATAGCCCCAG-3′ and the reverse primer was 5′-CTGGGGCTATCGTCGCACGCGGG TCTCTTGCTGGG- 3′.

The SAMHD1(15-626)-FLAG plasmid was created by amplifying the gene from pLPCX-SAMHD1-FLAG with the following primers: forward primer was 5′- GCG GAA TTC ACC ATG TGC GAC GAT AGC CCC AGA ACA C and reverse primer was 5′- GCGC ATC GAT TCA CTT GTC GTC GTC GTC CTT GTA GTC CAT AGG GTC GTC CTT AAA C. PCR products were digested and cloned into the EcoRI and ClaI sites of pLPCX.

### Antibodies

Monoclonal antibodies against SAMHD1 were from Abnova. Anti-FLAG rabbit polyclonal, anti-GAPDH rabbit polyclonal, anti-rabbit-Cy3, anti-mouse Alexa 594, and anti-rabbit Alexa 488 were obtained from Sigma. Anti-GFP rabbit polyclonal antibodies were purchased from Clontech. Anti-fibrillarin monoclonal antibodies were purchased from Cytoskeleton, Inc.

### Immunofluorescence microscopy

Transfections of cell monolayers were performed using Lipofectamine Plus reagent (Invitrogen), according to the manufacturer’s instructions. Transfected cells were incubated at 37 °C for 24 h, unless otherwise stated. For indirect immunofluorescence microscopy, cell monolayers grown on coverslips were transfected. Monolayers were washed twice with PBS and fixed for 15 min with 4% paraformaldehyde in PBS. Paraformaldehyde-fixed cells were washed twice with PBS, incubated for 4 min in permeabilizing buffer (0.5% Triton X-100 in PBS), and then blocked using PBS containing 2% bovine serum albumin for 1 h at room temperature. Subsequently, cells were incubated for 1 h at room temperature with primary antibodies in blocking buffer. After three washes with PBS, the cells were incubated for 30 min with secondary antibodies. Cellular nuclei were labeled with 1 μg/ml of DAPI (49, 69-diamidino-2-phenylindole) in PBS. Subsequently, samples were mounted for fluorescence microscopy by using the ProLong Antifade Kit (Molecular Probes, Eugene, OR). Images were obtained with a Zeiss Observer.Z1 microscope using a 63X objective, and deconvolution was performed using the software AxioVision V4.8.1.0 (Carl Zeiss Imaging Solutions).

### Protein analysis

Cellular proteins were extracted with radioimmunoprecipitation assay (RIPA) buffer, as previously described [36]. Detection of proteins by Western blotting was performed using anti-FLAG (Sigma), anti-GAPDH (Sigma), anti-SAMHD1 (Abnova), anti-GFP (clontech), and anti-rabbit and anti-mouse antibodies conjugated to Alexa Fluor 680(Li-Cor). Bands were detected by scanning blots with the Li-Cor Odyssey Imaging System using the 700 channel.

### Generation of cells stably expressing SAMHD1 variants

Retroviral vectors encoding wild-type SAMHD1, SAMHD1 fused to FLAG epitope or SAMHD1 mutants were created using the pLPCX vector (Clontech). Recombinant viruses were produced in 293T cells by cotransfecting the pLPCX plasmids with the pVPack-GP and pVPack-VSV-G packaging plasmids (Stratagene). The pVPack-VSV-G plasmid encodes the vesicular stomatitis virus G envelope glycoprotein, which allows efficient entry into a wide range of vertebrate cells [37]. Transduced HeLa and human monocytic U937 cells were selected in 5 μg/ml and 0.2 μg /ml puromycin, respectively.

### Infection with viruses expressing green fluorescent protein (GFP)

Recombinant HIV-1, SIV_mac_ and SIV_mac_ΔVpx expressing GFP were prepared as described [38]. All recombinant viruses were pseudotyped with the VSV-G glycoprotein. For infections, 5 x 10^4^ cells seeded in 24-well plates were incubated at 37°C with virus for 24 hours. Cells were washed and returned to culture for 48 hours, and the percentage of GFP-positive cells was determined by flow cytometry (Becton Dickinson). Viral stocks were titrated by serial dilution on Cf2Th cells to determine the concentration of infectious viruses.

### Preparation of monocyte-derived macrophages

Human monocytes were obtained from normal blood donor buffy coats by two-step gradient centrifugation followed by an additional step using the Monocyte Isolation kit II (Miltenyi Biotec) as previously described [39]. Macrophages were obtained by culturing monocytes for 7 days in RPMI 1640 (Invitrogen) supplemented with 10% FBS (Sigma) and autologous serum at a density of 1.5 x 10^5^/cm^2^.

### Infection of SAMHD1-silenced THP-1 cells expressing the different SAMHD1 variants

THP-1 cells stably expressing an shRNA targeting SAMHD1 and C-terminally FLAG- and HA- tagged shRNA-resistant SAMHD1 have been previously described[17]. SAMHD1 proteins harboring mutations K11A, R12A and R14A were generated on the shRNA-resistant SAMHD1 backbone in the previously described MMLV-based retroviral vector (pOZ-puro) [40]. Production of retroviral particles was achieved using the standard phosphate calcium procedure to transfect 293T cells (5μg pOZ construct, 2.5μg packaging plasmid and 2.5μg A-MLV envelope encoding plasmid). Silenced THP-1 cells were transduced or not with retroviral particles 48 hours prior to a 16 hours differentiation step using phorbol-12-myristate-13-acetate (PMA). Cells were subsequently infected with 30 ng of a VSV-G pseudotyped molecular clone of HIV-1 encoding the luciferase gene as a reporter. Luciferase activity was measured 24 hrs post infection and normalized for protein concentration.

### Vpx-induced degradation of SAMHD1

N-terminally FLAG- and HA-tagged Vpxmac251, VpxROD, VpxRCM-NG [17] and HIV-2B have been previously described [31]. HeLa cells were co-transfected with 0.250 μg SAMHD1 or SAMHD1-K11A together or not with 3μg Vpx alleles, using the jetPEI kit (Polyplus). Thirty-six hours post-transfection the cells were harvested; whole cell extracts prepared and analyzed by Western blotting using antibodies against FLAG or HA.

## Competing interests

The authors declare that they have no competing interests.

## Authors’ contributions

ABN performed experiments and helped with revision of the manuscript. JCV performed experiments and helped with revision of the manuscript. TEW performed experiments and helped with revision of the manuscript. NL performed, designed experiments and helped with revision of the manuscript. MB designed experiments and helped with revision of manuscript. JB helped with revision of manuscript. FDG designed experiments, wrote manuscript, and supervised the project. All authors read and approved the final manuscript.

## Supplementary Material

Additional file 1 **Intracellular distribution of SAMHD1 in differents types of cells. (A)** The intracellular distribution of endogenous SAMHD1 was studied in human monocyte derived macrophages (MDM), PMA-treated human THP-1 monocytic cells, and human HeLa cells. Cells were fixed and immunostained using antibodies against SAMHD1(red), as described in Materials and Methods. The cellular nuclei were stained using DAPI (blue). Image quantification for three independent experiments is shown in Table 1. **(B)** Localization of exogenously expressed SAMHD1 in HeLa cells. Transfected or transduced cells with plasmids expressing the indicated proteins and immunostained using antibodies against SAMHD1(red) or FLAG(red), as indicated. Similarly, the nuclei were stained with DAPI (blue). Image quantification for three independent experiments is shown in Table 1. **(C)** Expression of SAMHD1 in the indicated cells was analyzed by Western blotting using antibodies against SAMHD1 or FLAG. As a control, cell lysates were Western blotted using antibodies against GAPDH. **(D)** HeLa cells transfected with SAMHD1-FLAG were stained using antibodies against FLAG(red) and fibrilarin (green). DAPI was used to label the cellular nuclei. Similar results were obtained in three independent experiments, and a representative experiment is shown. Click here for file

Additional file 2 **Intracellular distribution of SAMHD1(15-626)-FLAG in HeLa cells. (A)** HeLa cells expressing the indicated SAMHD1 variant were fixed and immunostained using antibodies against FLAG(red), as described in Materials and Methods. The cellular nuclei were stained using DAPI (blue). Image quantification for three independent experiments is shown in Table 1. **(B)** Expression of the indicated SAMHD1 variant in HeLa cells was analyzed by Western blotting using antibodies against FLAG. As a control, cell lysates were Western blotted using antibodies against GAPDH. Similar results were obtained in three independent experiments and a representative experiment is shown. Click here for file

Additional file 3 **Intracellular distribution of the different Vpx alleles. (A)** HeLa cells were co-transfected with 0.25ug of SAMHD1 or SAMHD1-K11A together or not with 3 μg of the indicated HA tagged Vpx allele similar to the degradation experiments performed in Figure 5A. Thirty-six hours post-transfection the cells were fixed and stained using antibodies against SAMHD1 (green) and HA(red). In this experiment SAMHD1 was stained using a suboptimal concentration of antibody that do not stain the endogenous SAMHD1 from HeLa cells. The nuclei were stained with DAPI (blue). **(B)** Image quantification was performed by counting 200 Vpx-positive cells. Results are expressed as the number of Vpx-positive cells where co-staining of Vpx and SAMHD1 was observed (**Co-staining)**. Similarly, we show the number of Vpx-positive cells where SAMHD1 was not observed **(No Co-staining)**. This experiment was repeated three times and standard deviation is shown. Click here for file

Additional file 4 **SAMHD1 localization does not change upon infection of HIV-1. (A)** Human monocytic U937 cells stably expressing the indicated SAMHD1 variants were challenged by HIV-1-GFP virus(green) using an amount of virus that will infect ~40% of cells containing the empty vector pLPCX. Cells were fixed at the indicated hours post-infection (h.p.i.) and immunostained using antibodies against SAMHD1 (red), as described in Materials and Methods. The nuclei were stained with DAPI (blue). **(B)** Image quantification for three independent experiments is shown. **(C)** Similar challenges were incubated for forty-eight hours and infection was determined by measuring the percentage of GFP-positive cells. Similar results were obtained in three independent experiments and the standard deviation is shown. Click here for file
