# Supplementary material for: Role of SAMHD1 nuclear localization in restriction of HIV-1 and SIVmac
Source: Retrovirology. 2012 Jun 12;9:49. doi: 10.1186/1742-4690-9-49 (PMC3410799; doi:10.1186/1742-4690-9-49)
Supplement: Additional file 1 — Intracellular distribution of SAMHD1 in differents types of cells. (A) The intracellular distribution of endogenous SAMHD1 was studied in human monocyte derived macrophages (MDM), PMA-treated human THP-1 monocytic cells, and human HeLa cells. Cells were fixed and immunostained using antibodies against SAMHD1(red), as described in Materials and Methods. The cellular nuclei were stained using DAPI (blue). Image quantification for three independent experiments is shown in Table 1. (B) Localization of exogenously expressed SAMHD1 in HeLa cells. Transfected or transduced cells with plasmids expressing the indicated proteins and immunostained using antibodies against SAMHD1(red) or FLAG(red), as indicated. Similarly, the nuclei were stained with DAPI (blue). Image quantification for three independent experiments is shown in Table 1. (C) Expression of SAMHD1 in the indicated cells was analyzed by Western blotting using antibodies against SAMHD1 or FLAG. As a control, cell lysates were Western blotted using antibodies against GAPDH. (D) HeLa cells transfected with SAMHD1-FLAG were stained using antibodies against FLAG(red) and fibrilarin (green). DAPI was used to label the cellular nuclei. Similar results were obtained in three independent experiments, and a representative experiment is shown. [file 1742-4690-9-49-S1.pdf]

Additional File 1. Intracellular distribution of SAMHD1 in differents types of cells. (A) The intracelular distribution of endogenous SAMHD1 was studied in human monocyte derived macrophages (MDM), PMA-treated human THP-1 monocytic cells, and human HeLa cells. Cells were fixed and immunostained using antibodies against SAMHD1(red), as described in Materials and Methods. The cellular nuclei was stained using DAPI (blue). Image quantification for three indepedent experiments is shown in Table 1. **(B)** Localization of exogenously expressed SAMHD1 in HeLa cells. Transfected or transduced cells with plasmids expressing the indicated proteins and immunostained using antibodies against SAMHD1(red) or FLAG(red), as indicated. Similarly, the nuclei were stained with DAPI (blue). Image quantification for three indepedent experiments is shown in Table 1. **(C)** Expression of SAMHD1 in the indicated cells was analyzed by Western blotting using antibodies against SAMHD1 or FLAG. As a control, cell lysates were Western blotted using antibodies against GAPDH. **(D)** Hela cells transfected with SAMHD1-FLAG were stained using antibodies against FLAG(red) and fibrilarin (green). DAPI was used to label the cellular nuclei. Similar results were obtained in three independent experiments and a representative experiment is shown.

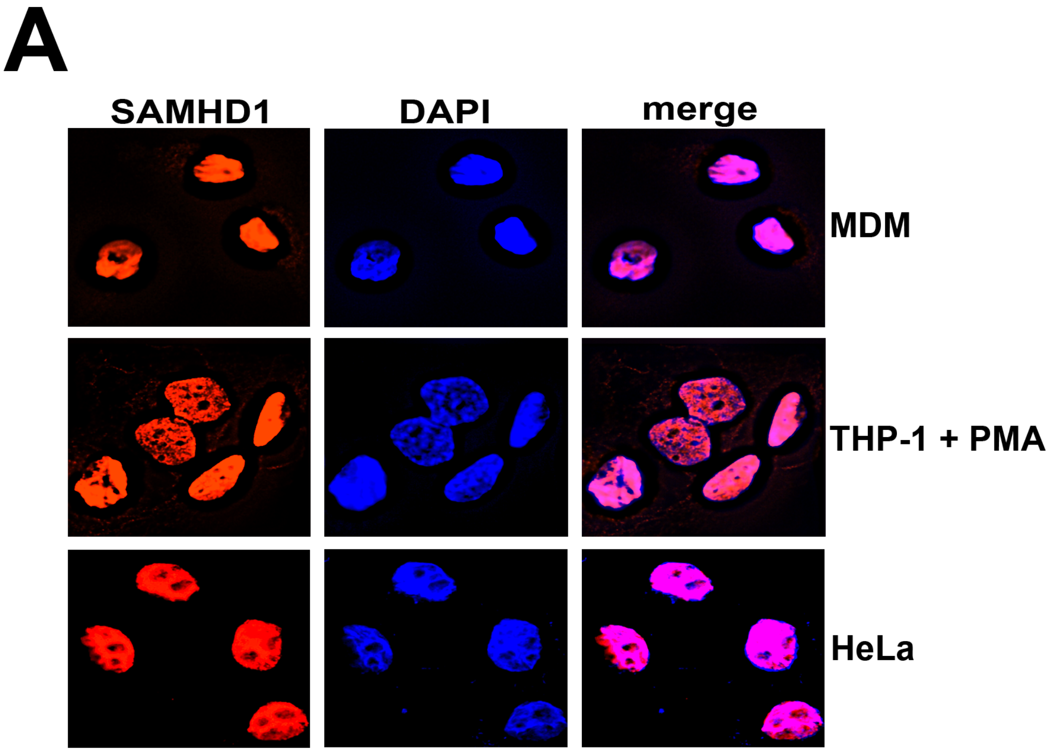

AF1

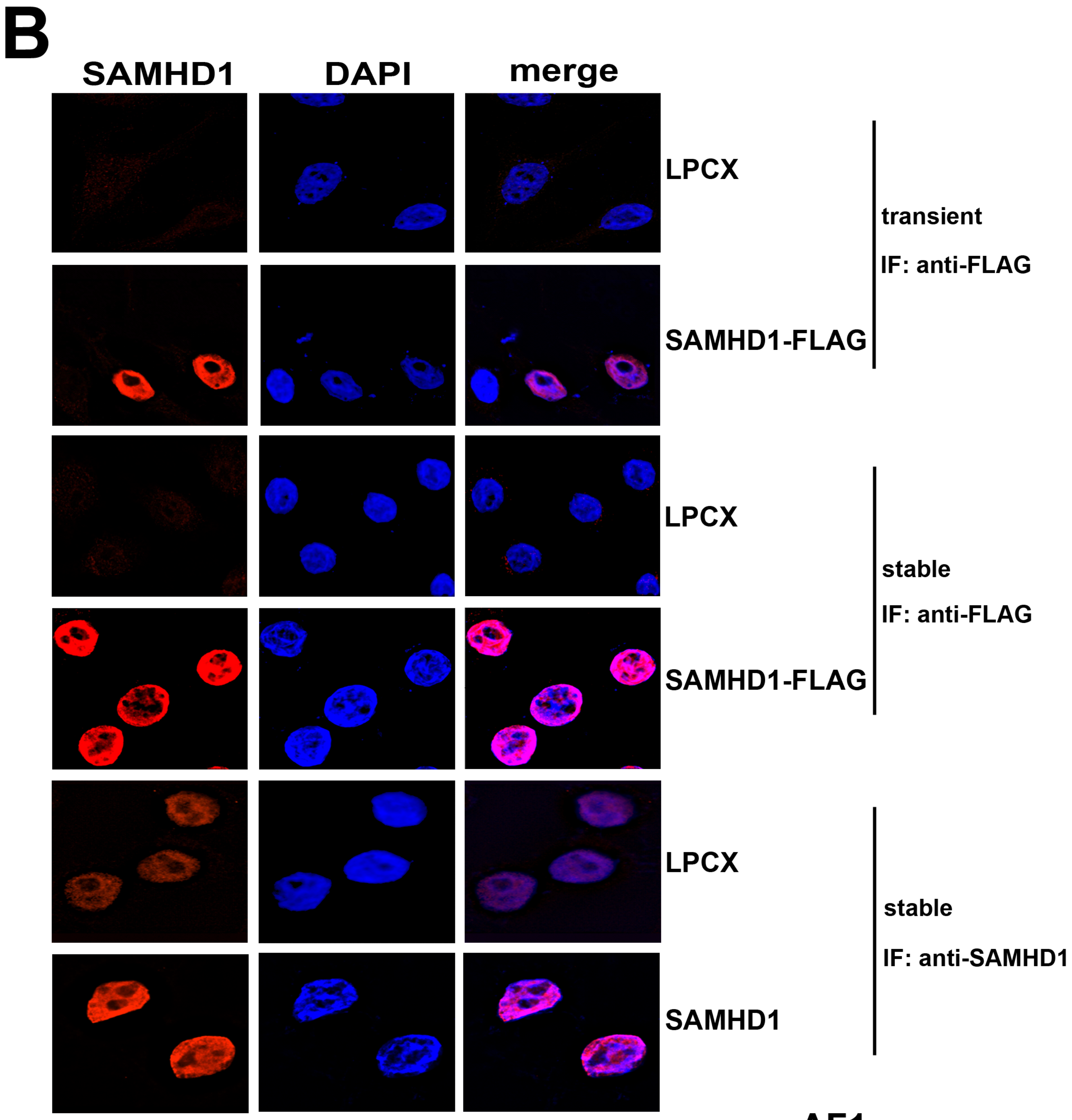
B
SAMHD1
DAPI
merge
LPCX
SAMHD1-FLAG
transient
IF: anti-FLAG
LPCX
SAMHD1-FLAG
stable
IF: anti-FLAG
LPCX
SAMHD1
stable
IF: anti-SAMHD1

AF1

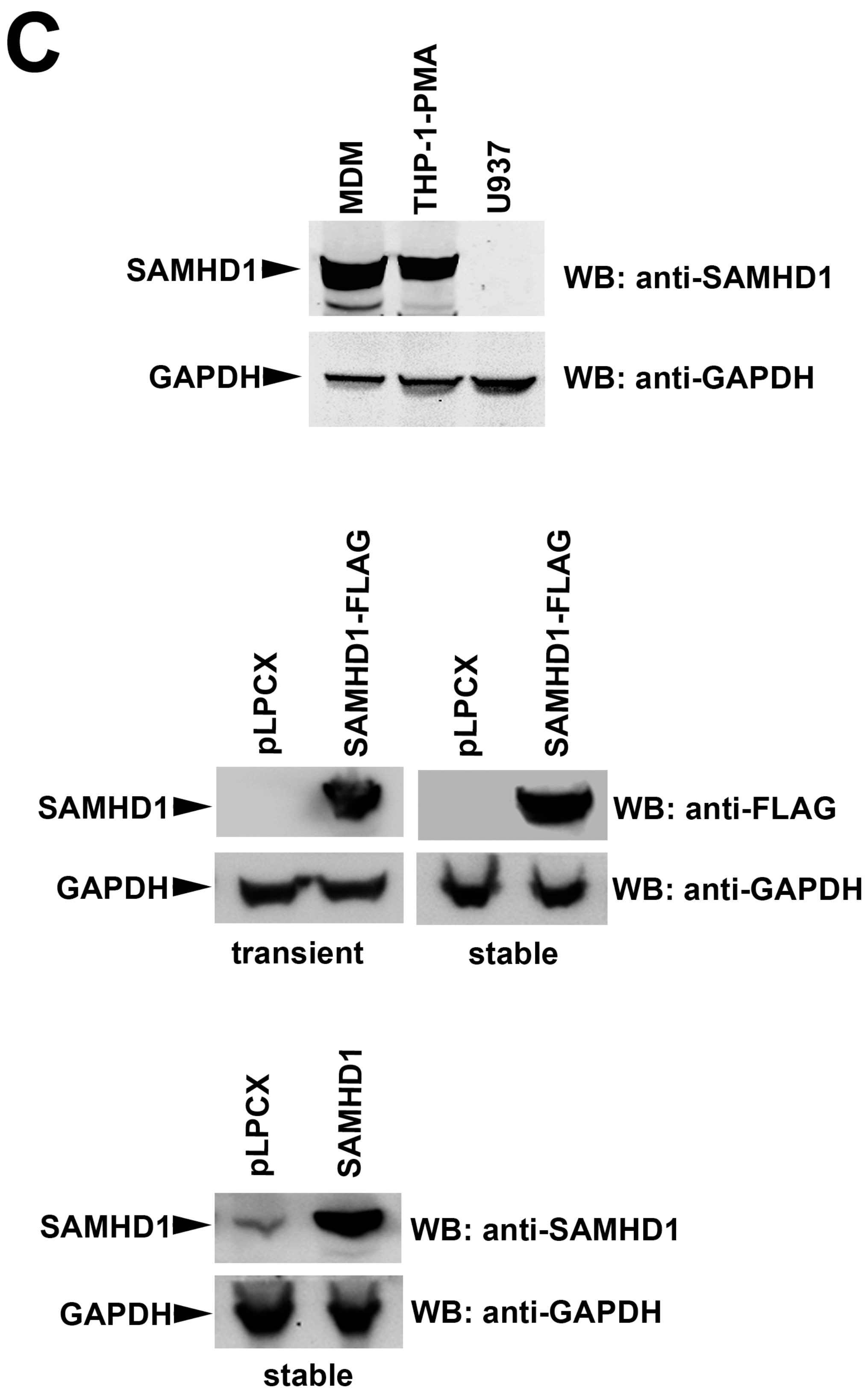
C
MDM
THP-1-PMA
U937
SAMHD1
WB: anti-SAMHD1
GAPDH
WB: anti-GAPDH
pLPCX
SAMHD1-FLAG
pLPCX
SAMHD1-FLAG
SAMHD1
WB: anti-FLAG
GAPDH
WB: anti-GAPDH
transient
stable
pLPCX
SAMHD1
SAMHD1
WB: anti-SAMHD1
GAPDH
WB: anti-GAPDH
stable

AF1

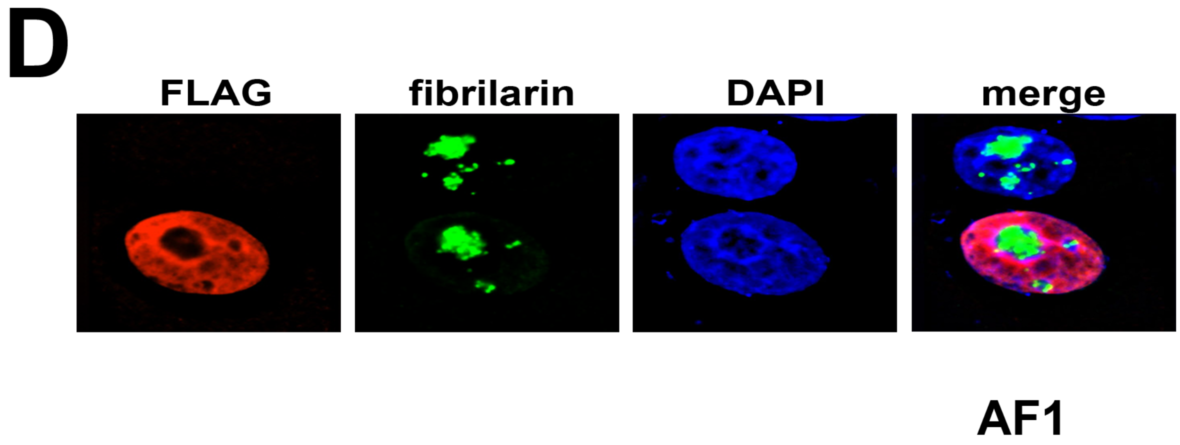
D
FLAG
fibrilarin
DAPI
merge
AF1
