# Supplementary material for: Role of SAMHD1 nuclear localization in restriction of HIV-1 and SIVmac
Source: Retrovirology. 2012 Jun 12;9:49. doi: 10.1186/1742-4690-9-49 (PMC3410799; doi:10.1186/1742-4690-9-49)
Supplement: Additional file 2 — Intracellular distribution of SAMHD1(15-626)-FLAG in HeLa cells. (A) HeLa cells expressing the indicated SAMHD1 variant were fixed and immunostained using antibodies against FLAG(red), as described in Materials and Methods. The cellular nuclei were stained using DAPI (blue). Image quantification for three independent experiments is shown in Table 1. (B) Expression of the indicated SAMHD1 variant in HeLa cells was analyzed by Western blotting using antibodies against FLAG. As a control, cell lysates were Western blotted using antibodies against GAPDH. Similar results were obtained in three independent experiments and a representative experiment is shown. [file 1742-4690-9-49-S2.pdf]

Additional File 2. Intracellular distribution of SAMHD1(15-626)-FLAG in HeLa cells. (A) HeLa cells expressing the indicated SAMHD1 variant were fixed and immunostained using antibodies against FLAG(red), as described in Materials and Methods. The cellular nuclei was stained using DAPI (blue). Image quantification for three indepedent experiments is shown in Table 1. **(B)** Expression of the indicated SAMHD1 variant in HeLa cells was analyzed by Western blotting using antibodies against FLAG. As a control, cell lysates were Western blotted using antibodies against GAPDH. Similar results were obtained in three independent experiments and a representative experiment is shown.

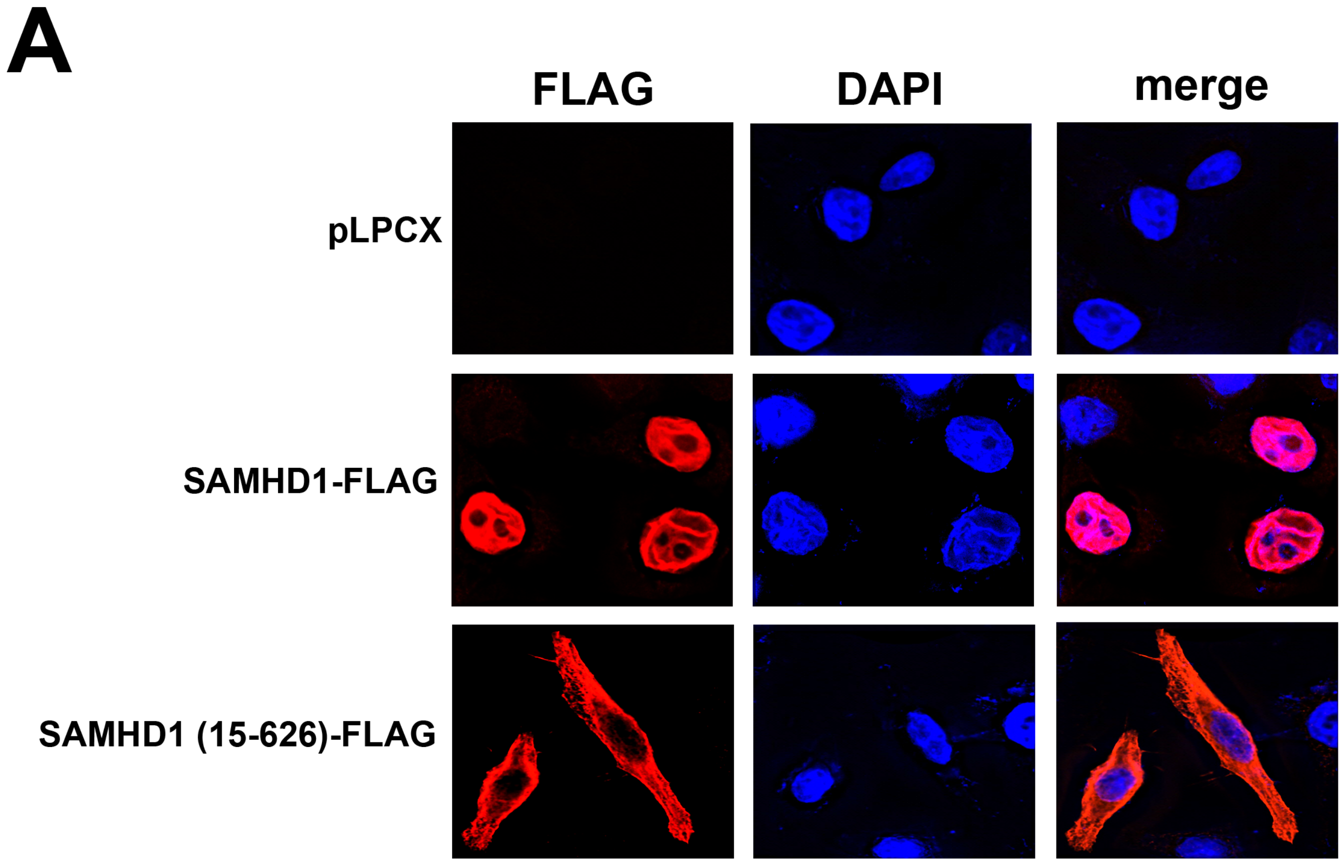

AF2A

B

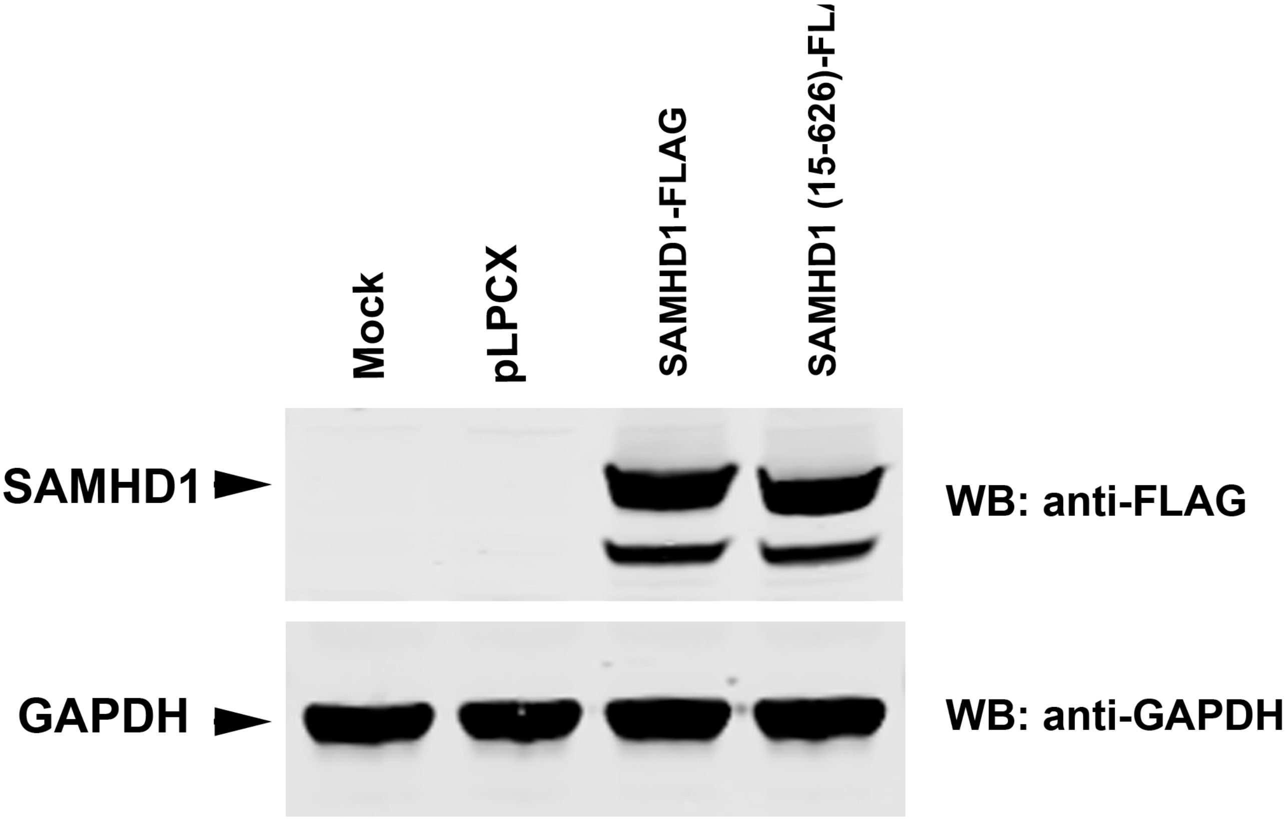
Mock
pLPCX
SAMHD1-FLAG
SAMHD1 (15-626)-FL
SAMHD1
GAPDH
WB: anti-FLAG
WB: anti-GAPDH

AF2B
