# Supplementary material for: Role of SAMHD1 nuclear localization in restriction of HIV-1 and SIVmac
Source: Retrovirology. 2012 Jun 12;9:49. doi: 10.1186/1742-4690-9-49 (PMC3410799; doi:10.1186/1742-4690-9-49)
Supplement: Additional file 3 — Intracellular distribution of the different Vpx alleles. (A) HeLa cells were co-transfected with 0.25ug of SAMHD1 or SAMHD1-K11A together or not with 3 μg of the indicated HA tagged Vpx allele similar to the degradation experiments performed in Figure 5A. Thirty-six hours post-transfection the cells were fixed and stained using antibodies against SAMHD1 (green) and HA(red). In this experiment SAMHD1 was stained using a suboptimal concentration of antibody that do not stain the endogenous SAMHD1 from HeLa cells. The nuclei were stained with DAPI (blue). (B) Image quantification was performed by counting 200 Vpx-positive cells. Results are expressed as the number of Vpx-positive cells where co-staining of Vpx and SAMHD1 was observed (Co-staining). Similarly, we show the number of Vpx-positive cells where SAMHD1 was not observed (No Co-staining). This experiment was repeated three times and standard deviation is shown. [file 1742-4690-9-49-S3.pdf]

Additional File 3. Intracellular distribution of the different Vpx alleles. (A) HeLa cells were co-transfected with 0.25ug of SAMHD1 or SAMHD1-K11A together or not with 3 µg of the indicated HA tagged Vpx allele similar to the degradation experiments performed in Figure 5A. Thirthy-six hours post-transfection the cells were fixed and stained using antibodies against SAMHD1 (green) and HA(red). In this experiment SAMHD1 was stained using a suboptimal concentration of antibody that do not stain the endogenous SAMHD1 from HeLa cells. The nuclei were stained with DAPI (blue). **(B)** Image quantification was performed by counting 200 Vpx-positive cells. Results are expressed as the number of Vpx-positive cells where co-staining of Vpx and SAMHD1 was observed(**Co-staining)**. Similarly, we show the number of Vpx-positive cells where SAMHD1 was not observed **(No Co-staining)**. This experiment was repeated three times and standard deviation is shown.

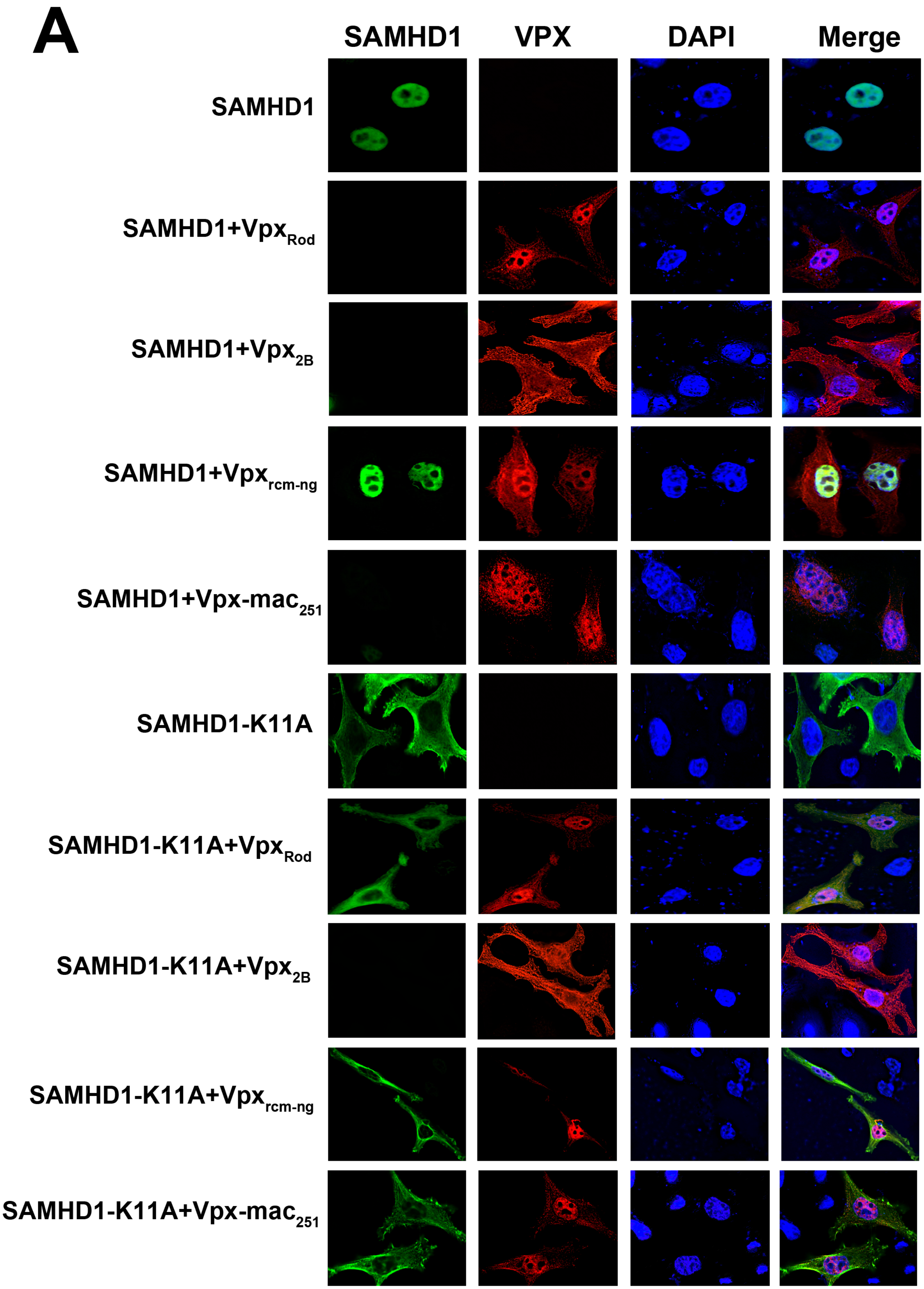
A
SAMHD1
VPX
DAPI
Merge
SAMHD1
SAMHD1+Vpx$_{Rod}$
SAMHD1+Vpx$_{2B}$
SAMHD1+Vpx$_{rcm\text{-}ng}$
SAMHD1+Vpx-mac$_{251}$
SAMHD1-K11A
SAMHD1-K11A+Vpx$_{Rod}$
SAMHD1-K11A+Vpx$_{2B}$
SAMHD1-K11A+Vpx$_{rcm\text{-}ng}$
SAMHD1-K11A+Vpx-mac$_{251}$

AF3A

B

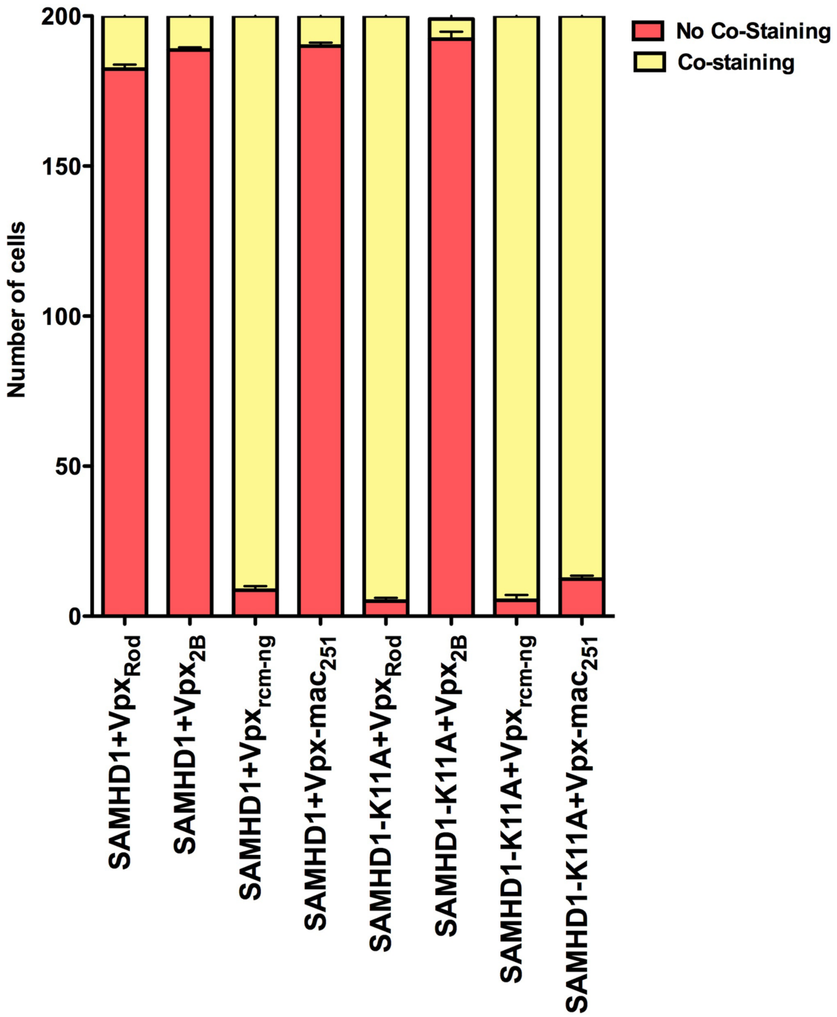
200
150
100
50
0
Number of cells
No Co-Staining
Co-staining
SAMHD1+Vpx$_{Rod}$
SAMHD1+Vpx$_{2B}$
SAMHD1+Vpx$_{rcm-ng}$
SAMHD1+Vpx-mac$_{251}$
SAMHD1-K11A+Vpx$_{Rod}$
SAMHD1-K11A+Vpx$_{2B}$
SAMHD1-K11A+Vpx$_{rcm-ng}$
SAMHD1-K11A+Vpx-mac$_{251}$

AF3B
