# Supplementary material for: Role of SAMHD1 nuclear localization in restriction of HIV-1 and SIVmac
Source: Retrovirology. 2012 Jun 12;9:49. doi: 10.1186/1742-4690-9-49 (PMC3410799; doi:10.1186/1742-4690-9-49)
Supplement: Additional file 4 — SAMHD1 localization does not change upon infection of HIV-1. (A) Human monocytic U937 cells stably expressing the indicated SAMHD1 variants were challenged by HIV-1-GFP virus(green) using an amount of virus that will infect ~40% of cells containing the empty vector pLPCX. Cells were fixed at the indicated hours post-infection (h.p.i.) and immunostained using antibodies against SAMHD1 (red), as described in Materials and Methods. The nuclei were stained with DAPI (blue). (B) Image quantification for three independent experiments is shown. (C) Similar challenges were incubated for forty-eight hours and infection was determined by measuring the percentage of GFP-positive cells. Similar results were obtained in three independent experiments and the standard deviation is shown. [file 1742-4690-9-49-S4.pdf]

Additional File 4. SAMHD1 localization does not change upon infection of HIV-1. (A) Human monocytic U937 cells stably expressing the indicated SAMHD1 variants were challenged by HIV-1-GFP virus(green) using an amount of virus that will infect ~40% of cells containing the empty vector pLPCX. Cells were fixed at the indicated hours post-infection (h.p.i.) and immunostained using antibodies against SAMHD1 (red), as described in Materials and Methods. The nuclei were stained with DAPI (blue). **(B)** Image quantification for three independent experiments is shown. **(C)** Similar challenges were incubated for forty-eight hours and infection was determined by measuring the percentage of GFP-positive cells. Similar results were obtained in three independent experiments and the standard deviation is shown.

A

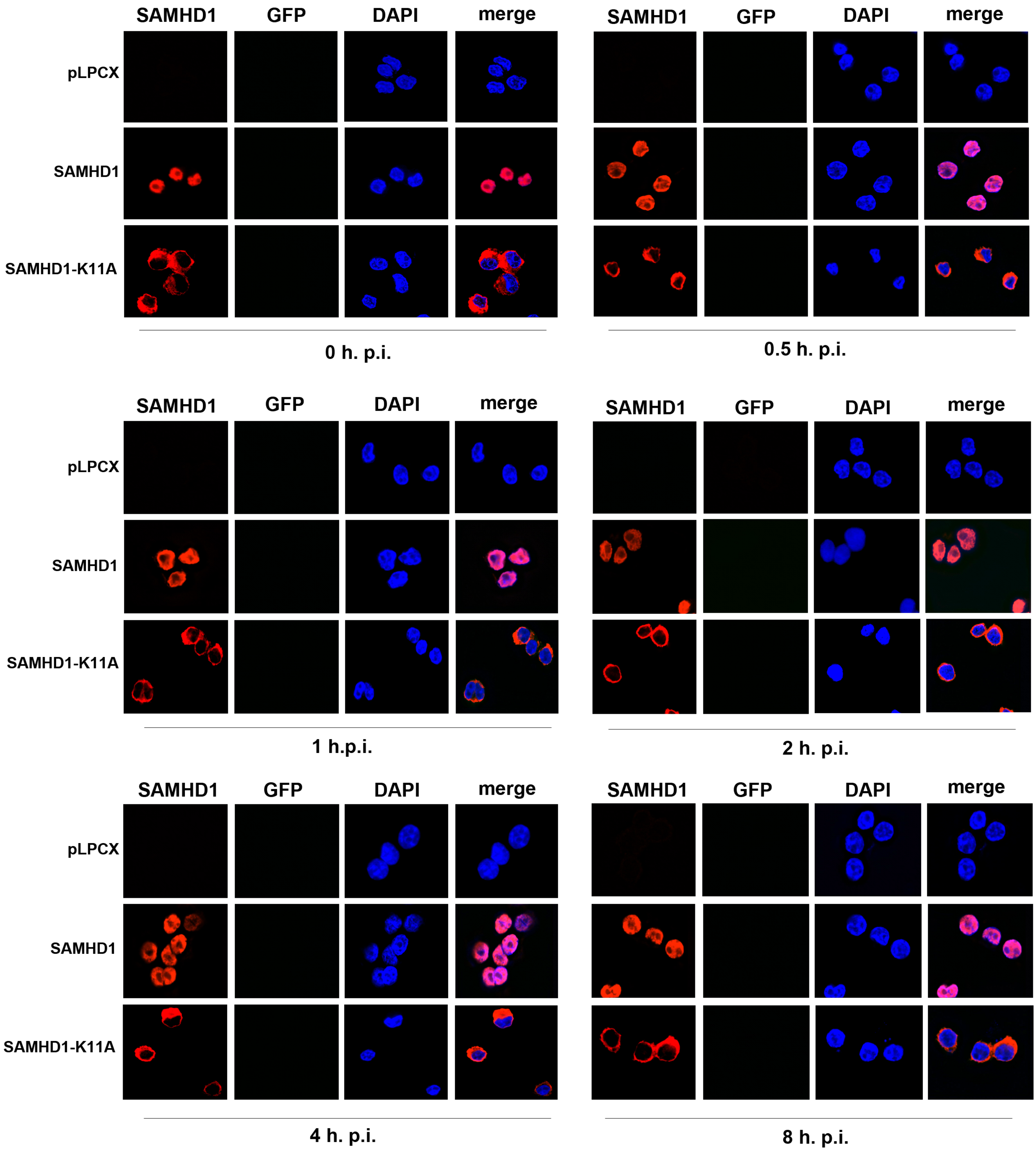
SAMHD1
GFP
DAPI
merge
pLPCX
SAMHD1
SAMHD1-K11A
0 h. p.i.
0.5 h. p.i.
1 h.p.i.
2 h. p.i.
4 h. p.i.
8 h. p.i.

AF4A

A

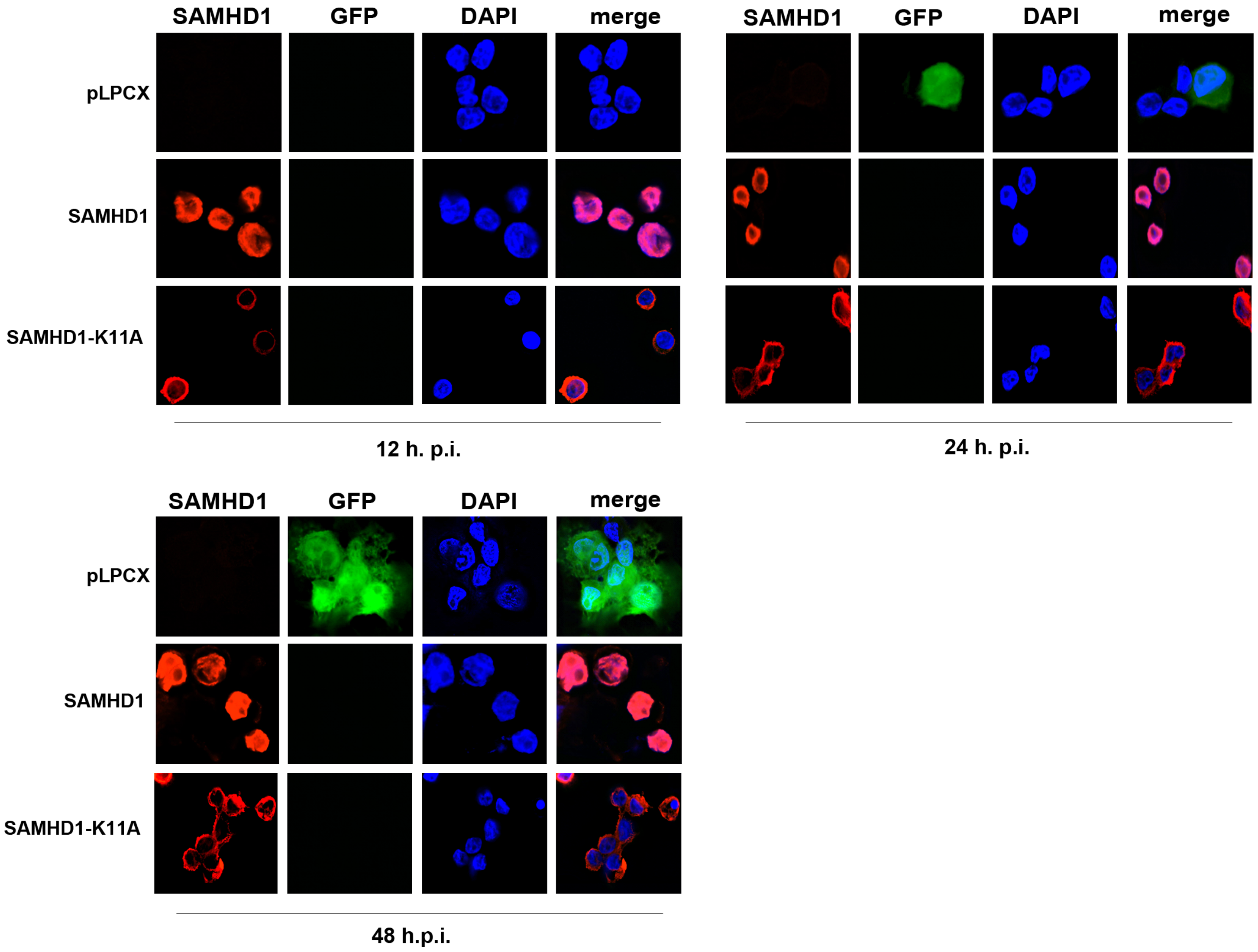
SAMHD1
GFP
DAPI
merge
pLPCX
SAMHD1
SAMHD1-K11A
12 h. p.i.
24 h. p.i.
48 h.p.i.

AF4A

B

		Experiment 1			Experiment 2			Experiment 3		
SAMHD1 Variant	HIV-1 Hours Post-infection(h.p.i)	Exclusively nuclear	Exclusively cytoplasm	Throughout cell	Exclusively nuclear	Exclusively cytoplasm	Throughout cell	Exclusively nuclear	Exclusively cytoplasm	Throughout cell
SAMHD1	0	195	0	5	192	0	8	196	0	4
SAMHD1 K11A	0	0	197	3	0	194	6	0	190	10
SAMHD1	0.5	193	0	7	191	0	9	188	0	12
SAMHD1 K11A	0.5	0	189	11	0	194	6	0	190	10
SAMHD1	1	190	0	10	195	0	5	189	0	11
SAMHD1 K11A	1	0	190	10	0	191	9	0	192	8
SAMHD1	2	189	0	11	195	0	5	190	0	10
SAMHD1 K11A	2	0	192	8	0	196	4	0	186	14
SAMHD1	4	196	0	4	194	0	6	196	0	4
SAMHD1 K11A	4	0	194	6	0	192	8	0	189	11
SAMHD1	8	192	0	8	189	0	11	197	0	3
SAMHD1 K11A	8	0	192	8	0	190	10	0	188	12
SAMHD1	12	196	0	4	187	0	13	198	0	2
SAMHD1K11A	12	0	189	11	0	192	8	0	195	5
SAMHD1	24	195	0	5	190	0	10	197	0	3
SAMHD1 K11A	24	0	196	4	0	193	7	0	189	11
SAMHD1	48	192	0	8	186	0	14	193	0	7
SAMHD1 K11A	48	0	188	12	0	197	3	0	194	6

AF4B

C

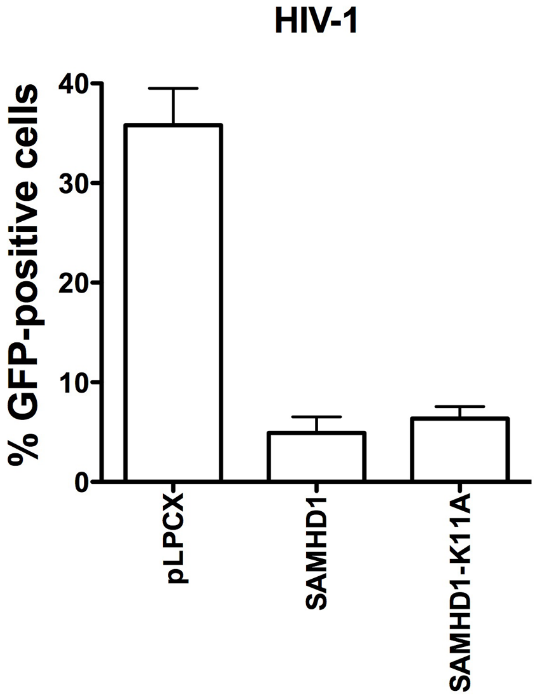
HIV-1
% GFP-positive cells
40
30
20
10
0
pLPCX
SAMHD1
SAMHD1-K11A

AF4C
